# Aberrant Promoter Hypermethylation of *RASSF1a* and *BRCA1 *in Circulating Cell-Free Tumor DNA Serves as a Biomarker of Ovarian Carcinoma

**DOI:** 10.31557/APJCP.2019.20.10.3001

**Published:** 2019

**Authors:** Sandeep Kumar S, Shalini N Swamy, C S Premalatha, V R Pallavi, Ramesh Gawari

**Affiliations:** 1 *Department of Biochemistry,*; 2 *Department of Pathology,*; 3 *Department of Gynaeconcology, Kidwai Memorial Institute of Oncology, Bangalore, Karnataka, India.*

**Keywords:** Ovarian cancer, promoter hypermethylation, biomarker, circulating tumor DNA, RASSF1a, BRCA1

## Abstract

**Objective:**

Ovarian cancer is one of the leading causes of cancer deaths in women. Ovarian cancer is diagnosed at the late stages and generally relapses within 12-14 months of cytoreductive surgery. This is attributed to lack of precise molecular detection methodologies to detect and track the disease. Epigenetic alteration such as aberrant promoter hypermethylation is an important early event that occurs during cancer development and progression. This study focuses on development of a minimally invasive methylation marker that could be used for detection and prognosis of ovarian cancer patients.

**Methods:**

Aberrant promoter hypermethylation of *RASSF1a *and *BRCA1* was assessed in circulating DNA of 72 EOC patients using methylation-specific PCR. The findings were correlated with various clinicopathological parameters. Statistical analysis was done using the Fisher exact test and chi-square test.

**Results:**

The aberrant methylation patterns of *RASSF1a *and *BRCA1* was identified to be present in the cancerous samples. A total of 31.9 % and 56.9% methylation was observed for *RASSF1a *and *BRCA1* respectively. A striking 50% methylation of *BRCA1* was identified in the benign sample cohort, which marks the significance of assessing the hypermethylation pattern to detect cancer at its early stages. Methylation of the two tumor suppressor genes was evident across various stages and grades of ovarian tumors suggesting that this could also help as a prognostic marker.

**Conclusion:**

The results of the current study hold significance since the hypermethylation patterns can be identified in the cell-free circulating tumor DNA from a small volume of blood plasma and is a simple and minimally-invasive method. Assessment of hypermethylation patterns of a panel of TSG along with the existing screening markers could aid in better diagnosis and management of the disease. It could also aid in designing specifically tailored treatment strategies to fight the disease.

## Introduction

Ovarian cancer is the third leading gynecological cancer in India and has a very high mortality rate compared to other cancers (Globacon., 2018). Ovarian cancer is mostly asymptomatic at the early stages and the patients do not report with any major clinical symptoms. The high mortality rate of ovarian cancer is attributed to the diagnosis of the disease at stage III or IV, where the cancer would have already metastasized to distant sites. The five-year survival rate of ovarian cancers diagnosed at stage III and IV is about 42% and 26% as against 93% when it is diagnosed at the early stages (Torre et al., 2018). 

Despite advances in diagnosis, it has not been possible to detect the disease at the early stage. There is an increasing need to develop new and novel detection methodologies to identify the disease at the early stages.

**Table 1:** Methylation Specific PCR Conditions

Gene	Initial Denaturation	Cycling Stage (35 cycles)			Final Extension
		Denaturation	Annealing	Extension	
*RASSF1a*	95^0^C	95^0^C	60^0^C(M)/58^0^C(UM)	72^0^C	72^0^C
Time	10 nims	30 sec	30 sec	30 sec	7 mins
*BRCA1*	95^0^C	95^0^C	65^0^C(M)/61^0^C(UM)	72^0^C	72^0^C
Time	5 mins	30 sec	30 sec	30 sec	5 min

**Table 2:** Methylation Frequencies for *RASSF1a*

Genes	Tumor type			
	Malignant (53)	LMP (07)	Benign (12)	Normal (15)
*RASSF1A *(M)	20/53 (38%)	1/7 (14%)	2/12 (17%)	0/15 (0%)
*RASSF1A*(U)	33/53 (62%)	6/7 (86%)	10/12 (83%)	15/15 (100%)
p Value	0.0033**	0.318	0.188	

**Table 3:** Methylation Frequencies for *BRCA1*

Genes	Tumor type			
	Malignant (53)	LMP(07)	Benign (12)	Normal (15)
*BRCA1* (M)	33/53(62%)	2/7(29%)	6/12(50%)	0/15 (0%)
*BRCA1*(U)	20/53(38%)	5/7(71%)	6/12(50%)	15/15(100%)
pValue	0.0001***	0.0909	0.0031**	

**Figure 1:**
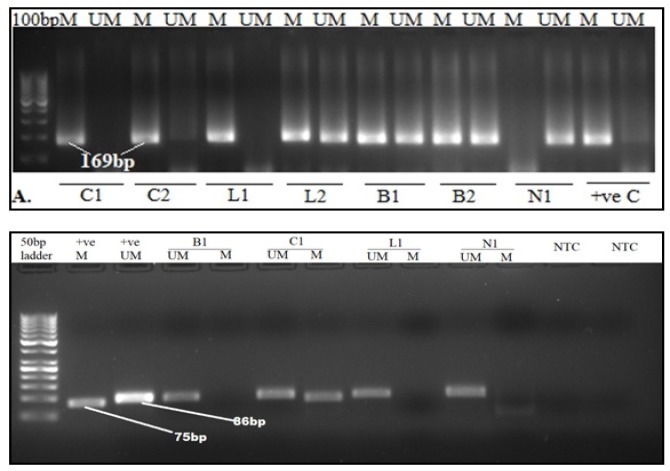
A and B, Representative Agarose gel images of RASSF1a and BRCA1. 2.5% Agarose gel depicting the products of MSP analysis of (A) RASSF1A and (B) BRCA1 genes in ovarian cancer patients. PCR products in the lanes labeled ‘M’ represent the methylated alleles and the lanes labeled ‘UM’ represent the unmethylated alleles. Universally methylated HeLa genomic DNA was used as a positive (+ve) control and peripheral-blood derived DNA from normal non-carcinoma subjects were used as a positive control for unmethylated alleles. PCR products in lane UM and M indicate the presence of an unmethylated and methylated allele. C1-2(carcinomas), B1-2 (benign adenomas), L1-2 (low malignant potential tumors) and N1 (non-cancer sample)

The onset of the tumor is attributed to several genetic and epigenetic alterations. One such epigenetic alteration that is known to occur in the early stages of tumor development is the aberrant hypermethylation of the CpG island in gene promoters. This aberrant hypermethylation is known to cause silencing of several tumor suppressor genes, thereby contributing to malignant transformation. Aberrant hypermethylation of TSGs can be studied in the cell-free circulating DNA of cancer patients.

Detection of molecular changes in liquid biopsies such as peripheral blood is gaining attention due to its advantages over tumor biopsies. Use of peripheral blood to detect molecular changes that occur during cancer progression holds a significant promise as a minimally invasive marker for detection and monitoring of the disease at any stage during therapy. 

Cell-free DNA from the tumor cells is shed into the bloodstream during the course of tumor progression. Epigenetic alterations such as aberrant promoter hypermethylation patterns can be identified in the cell-free circulating DNA of ovarian cancer patients and therefore, would allow for the real-time monitoring of the disease. 

This study aims at studying the aberrant promoter hypermethylation of two tumor suppressor genes, *RASSF1a *and *BRCA1*, known to be involved in ovarian carcinoma pathogenesis.

RAS associated domain family 1 A (RASSF1a), is a tumor suppressor gene located on chromosome 3p21.3 and is known to be involved in cell cycle regulation. *RASSF1A *falls into the category of the genes frequently silenced by methylation rather than mutation events (Chen H et al., 2006). Aberrant promoter hypermethylation of *RASSF1a *has been reported in various malignancies ranging from a frequency of 99% in the tumor to 0% in normal tissues (Donninger et al., 2007). 

Breast cancer susceptibility gene 1 (*BRCA1*), is a gene that is located on chromosome 17q12.21 mainly involved in the maintenance of genome stability and effective DNA repair mechanism (Zhang et al.,2015; Koukoura et al., 2014). Effect of genetic and epigenetic alterations in the *BRCA1* gene is attributed in many malignancies including breast and ovarian cancers (Esteller et al., 2000). *BRCA1* methylation frequencies have been reported to be ranging from 10-89% in ovarian cancers (Prieske e3et al., 2017).

In the present study, we have assessed the aberrant promoter hypermethylation status of *RASSF1a *and *BRCA1* in the cell-free circulating DNA (cfc DNA) of ovarian carcinoma patients using methylation-specific PCR.

## Materials and Methods

*Patients and sample collection*

Aberrant promoter hypermethylation was assessed in 72 epithelial ovarian carcinoma (EOC) patients and 15 (age matched) healthy subjects. Preoperative blood sample was obtained from all the subjects after obtaining written informed consent. The study has been approved by the Institutional Scientific Review Board and the Medical Ethics Committee.

*Blood collection and processing*

3ml of pre-operative blood sample was collected from the patients in a BD vacutainer (spray coated K2EDTA tubes). The blood was immediately centrifuged at 3,000 rpm for 10 mins to separate plasma. The plasma samples were stored at -80^o^C until DNA extraction.

*DNA Extraction*

The plasma sample was thawed on ice and incubated with Proteinase K (10 mg/ml) and 0.5% SDS overnight at 37^o^C. Post incubation the plasma samples were subjected to Phenol: Chloroform extraction and the DNA were precipitated with 10 mol/L ammonium acetate and 2µl of glycogen. The precipitated DNA was washed with 70% ethanol and eluted with 10µl of TE buffer. DNA aliquots were stored at -20^o^C until bisulfite modification.

*Analysis of DNA Methylation by Bisulfite modification*

DNA extracted from the plasma samples was subjected to Sodium Bisulfite modification using EZ DNA Methyl Lightening kit TM (Zymo Research, CA, USA) following the manufacturer’s protocol. Modified DNA was eluted in a final volume 10µl.

Promoter hypermethylation was assessed performing Methylation specific PCR using specific primers adapted from Honorio S et al., (2003) and Esteller M et al., (2000) (*RASSF1A Methylated forward* -5’-GGGTTTTGCGAGAGCGCGT-3’, *RASSF1A Methylated reverse*-5’-GCTAACAAACGCGAACCG-3’, *RASSF1A Unmethylated forward-5’-*GGTTTTGTGAGAGTGTGTTTAGT-3’, *RASSF1A Unmethylated reverse-*5’CACTAACAAACACAAACCAAACA-3’; *BRCA1* Methylated forward-5’-GGGTTTTGCGAGAGCGCGT-3’, *BRCA1* *Methylated reverse-5’*-AAAACTCAACGAACTCACGCCG-3’, *BRCA1* *Unmethylated forward-5’-*TTGGTTTTTGTGGTAATGGAAAAGTGT-3’, *BRCA1* *Unmethylated reverse*-5’-CAAAAAATCTCAACAAACTCACACCA-3’). The cycling conditions are mentioned in Table1.

Universally Methylated HeLa Genomic DNA (New England Biolabs Inc, England) was used as a positive control for the methylated allele. Peripheral blood-derived DNA from healthy non-cancer patients was used as a control for unmethylated allele and no template control served as a negative control.10µl of the PCR product was loaded on to a 2.5% Agarose gel and visualized by staining with Ethidium Bromide. 

*Measurement of CA125 and CEA*

Cancer antigen 125 (CA125) and Carcinoembryonic antigen (CEA) levels were estimated for all the 72 EOC patients and normal subjects included in the study. CA125 and CEA were estimated using the Elecsys CA125 and CEA diagnostic kits procured from Roche, Germany and quantified on Cobas e411TM auto analyzer (Roche Diagnostics, Germany). A CA125 value of 0-35U/ml and a CEA value between 0-7.5ng/ml were considered normal. 

Statistical analysis: The methylation status of *RASSF1A *and *BRCA1* was correlated with clinicopathological characteristics. Chi-square test or Fisher Exact test was used to analyze the data. All the data were statistically analyzed using the SPSS software version 21.0. A p-value of less than 0.05 was considered to hold a statistical significance.

## Results

*Methylation frequency of RASSF1a and BRCA1 in cfc DNA*

Aberrant promoter methylation was assessed in 15 normal samples and 72 tumor samples which included malignant (n=53), low malignant potential tumors (n=7) and benign samples (n=12).

20 out of 53 (38%) malignant samples, 1 out of the 7 (14%) low malignant potential tumors and 2 of the 12 (17%) benign samples showed positive for *RASSF1a *promoter methylation. The data is represented in Table 2. The representative gel image of the same is presented in Figure 1A. 

33 of the 53 (62%) malignant tumors, 2/7 (29%) low malignant potential tumors and 6 of the 12 (50%) of the benign samples showed positive for *BRCA1* promoter methylation. The summary of the same has been represented in Table 3. The representative gel image is presented in Figure 1B.

Methylation in both the genes was observed for 22 samples (30.5%) whereas, promoter hypermethylation in either one or both the genes were seen in 42/72 (58.3%) samples. Neither *BRCA1* nor *RASSF1A *genes showed methylation in the non-cancerous samples.

Presence of both methylated and unmethylated bands were observed in a few samples and this could be due to heterogeneity (presence of both cancer and normal cells). In such a case the samples are considered to be positive for methylation.

*Correlations of BRCA1 and RASSF1A Methylation with clinical factors*

All the samples included in this study belonged to the epithelial ovarian carcinoma and they were grouped based on menopausal status, histological subtype, FIGO stage, CA125, CEA levels and the presence or absence of ascites. The details of the same are summarized in table-3. Most of the tumors belonged to the high grade and advanced stages (38/53, 71.6%) and the most common histological subtype in the study was of the serous tumors (36/53, 67.9%).

The aberrant hypermethylation status of *BRCA1* and *RASSF1A *genes were assessed and its association with the available clinicopathological parameters were studied and summarized as in Table 4.

Based on the statistical analysis, a significant correlation was found to exist between the methylation status of *BRCA1* with histological type (p=0.042), FIGO stages (p<0.001), CA-125(p<0.001) and CEA (p<0.001) levels. *BRCA1* methylation did not show a correlation between the menopausal state and presence of ascites. On the other hand, *RASSF1A *methylation showed a significant correlation only with the menopausal status (p=0.041) and ascites levels (p=0.049). 

## Discussion

Genetic and epigenetic alterations are known to be implicated in the development of several cancers including ovarian cancer. Aberrant promoter hypermethylation is a common epigenetic alteration that causes inactivation of TSGs and is known to be an early event during carcinogenesis (Zuberi et al., 2014). Detection of these epigenetic alterations in cell free circulating DNA in blood and other body fluids is gaining importance due its ease of accessibility and non invasiveness.

In the present study, we have assessed the aberrant promoter hypermethylation of *RASSF1a *and *BRCA1* using methylation specific PCR. A methylation frequency of 56.9% was observed for *BRCA1*. Interestingly a 50% methylation frequency of *BRCA1* was observed in the benign sample cohort, suggesting that the methylation of *BRCA1* is indeed an early event in ovarian cancer. The methylation frequency of *BRCA1* showed a statistically significant correlation with histological subtype, FIGO staging, CA125 and CEA levels. In addition a methylation frequency of 31.9% was observed for *RASSF1a *and was found to be significantly associated with the menopausal status and CA125 levels. 

A similar study by De Caceres et al., (2004) have reported a methylation frequency of 18 % for *BRCA1* and 42% for *RASSF1a *in the serum of ovarian cancer patients. However there are other studies that have reported the methylation frequency of *BRCA1* and *RASSF1a *in the range of 16%-52% and 26%-66% respectively (Shilpa et al., 2014; Cathy et al.,2005; Bai et al., 2014; Jens et al., 2005; Prakash et al., 2005; Yoon et al., 2001; Bhagat et al., 2012; Matoo et al., 2013). But these finding are derived from the experiments performed using primary tumor tissue specimens and not circulating tumor DNA. Our study is the first report on aberrant hypermethylation of *RASSF1A *and *BRCA1* in cell-free circulating DNA of epithelial ovarian cancer from the Asian population.

The results of the present study and earlier reports are suggestive of the fact that the cell free circulating tumor DNA carries the representative epigenetic signatures of the primary tumor. Identification such aberrant epigenetic alterations that occur during tumorigenesis could thus serve as a promising molecular marker for the early detection of the disease. 

In conclusion, Since methylated genes appear to have superior specificity for cancer, highly specific DNA methylation marker of a panel of TSGs can be combined with the existing diagnostic modalities to increase the efficacy in diagnosis of ovarian cancer. Assessment of epigenetic alterations in cell-free DNA of cancer patients can serve as a minimally invasive and potential diagnostic and prognostic marker. 
